# Cost-utility analysis of certolizumab pegol versus alternative tumour necrosis factor inhibitors available for the treatment of moderate-to-severe active rheumatoid arthritis in Spain

**DOI:** 10.1186/s12962-015-0037-9

**Published:** 2015-06-09

**Authors:** Álvaro Hidalgo-Vega, Renata Villoro, Juan Antonio Blasco, Pablo Talavera, Belén Ferro, Oana Purcaru

**Affiliations:** Castilla-La Mancha University, Toledo, Spain; Max Weber Institute, Madrid, Spain; Lain Entralgo, Health Technology Assessment, Madrid, Spain; Medical Department, UCB Pharma, Madrid, Spain; Market Access-Pharmacoeconomic Department, UCB Pharma, Madrid, Spain; Global Market Access, UCB Pharma, Brussels, Belgium

## Abstract

**Background:**

Certolizumab pegol, a PEGylated tumour necrosis factor (TNF)-inhibitor, improves the clinical signs and symptoms of rheumatoid arthritis (RA) when used in combination with methotrexate or as monotherapy. This study evaluatedthe cost-utility of certolizumab pegol versusTNF-inhibitors plus methotrexate in the treatment of moderate-to-severe RA in Spain.

**Methods:**

A Markov cohort health state transition model was developed to evaluate the cost-utility (costs and quality-adjusted life years [QALYs]) of certolizumab pegol versus other TNF-inhibitors licensed in Spain in 2009. Efficacy was measured using the American College of Rheumatology (ACR) responses at 6 months, based on adjusted indirect comparisons from published clinical trials. Utilities were derived from EQ-5D data from certolizumab pegol RA clinical trials. Clinical history and resource use data came from published literature. Unit costs were taken from Spanish databases or published data (cost year 2009). Base case analyses were conducted from the payer perspective, with a lifetime horizon, 3.5 % annual discounting rates for costs and outcomes, and 3 % inflation rate for 2009 onwards. One-way sensitivity analyses were conducted.

**Results:**

The average lifetime costs for certolizumab pegol, etanercept, adalimumab (every 2 weeks and weekly) and infliximab (3 mg/kg and 5 mg/kg) in combination with methotrexate were €140,971, €141,197, €139,148, €164,741, €136,961 and €152,561, respectively. The QALYs gained were 6.578, 6.462, 6.430 (for both adalimumab doses), 6.430, and 6.318 (for both infliximab doses), respectively. At a €30,000/QALY willingness-to-pay threshold, certolizumab pegol plus methotrexate dominated adalimumab weekly, etanercept, and infliximab 5 mg/kg, and was cost-effective versus adalimumab every 2 weeks and infliximab 3 mg/kg (all with methotrexate), with estimated ICERs of €12,346/QALY and €15,414/QALY, respectively. Certolizumab pegol monotherapy was more cost-effective versus adalimumab, and less expensive with similar health gains versus etanercept (6.416 QALYs vs 6.492). Univariate analysis showed ICERs to be sensitive to changes in time horizon, ACR response time point, baseline Heath Assessment Questionnaire (HAQ) score, and rate of HAQ-disability index deterioration after discontinuing treatment.

**Conclusions:**

This analysis shows that certolizumab pegol is cost-effective compared with other TNF-inhibitors recommended in Spain for the treatment of RA.

## Background

Rheumatoid arthritis (RA) is a chronic inflammatory disease causing progressive joint destruction, deformity and disability. Although its exact aetiology is unknown, RA is believed to be an autoimmune disease stimulated by environmental factors in genetically susceptible individuals [1]. The prevalence in Spain is 0.5 % according to the EPISER study [2], with an incidence estimated at 8.3 cases per 100,000 by the Spanish Society of Rheumatology. The annual incidence of RA in adults in Spain is in the lower range for European countries, and comparable with those in other Mediterranean countries [3].

The aim of treatment is disease remission or the lowest disease activity possible. Standard treatment for RA patients in Spain with persistent disease in spite of aggressive management currently consists of disease-modifying antirheumatic drugs (DMARDs). In line with national guidelines, methotrexate, a small-molecule DMARD, is the first treatment choice in Spain for more than 80 % of patients with RA [4]. Biological DMARDs include tumour necrosis factor (TNF) inhibitors, e.g. certolizumab pegol, adalimumab, golimumab, infliximab and etanercept, which target TNFα, a proinflammatory cytokine believed to play a major role in the pathogenesis of RA [5]. TNF inhibitors or tocilizumab, an antibody directed against the interleukin-6 receptor, administered alone or in combination with methotrexate, are the first treatment option after small-molecule DMARDs [6, 7]. Other biological agents used in Spain are anakinra, abatacept and rituximab, which are used in patients with RA who do not respond to methotrexate and in patients with active RA despite treatment with TNF inhibitors [6, 7]. However, a significant proportion of patients has an unsatisfactory response to these treatments and continues to experience episodes of disease activity while receiving therapy [8–11].

Certolizumab pegol (Cimzia®, CZP) is a PEGylated Fc-free anti-TNF approved for adults with moderate to severe RA [12, 13]. It is administered by subcutaneous injection and has a relatively long elimination half-life, allowing administration once every 2 or 4 weeks. Certolizumab pegol demonstrated rapid and sustained improvements in physical function and signs and symptoms of RA, and relief in pain and fatigue and significant improvements in productivity at work and home and participation in social activities [14–17]. CZP is approved in Spain, either as monotherapy or in combination with methotrexate, for the treatment of moderate-to-severe, active RA in adult patients when the response to DMARDs, including methotrexate, has been inadequate [13].

Whilst TNF inhibitors have generally been shown to be cost-effective in the treatment of RA [18–26], data regarding the relative cost-effectiveness of the various TNF inhibitors are limited and there are few published economic evaluations for certolizumab pegol.

The aim of this study was to evaluate the cost utility of certolizumab pegol compared with other standard first-line TNF-inhibitor therapies licensed and marketed in Spain in 2009 (etanercept, adalimumab, infliximab), administered with or without methotrexate for the treatment of patients with moderate-to-severe RA who have had an inadequate response to methotrexate alone.

## Methods

### Cost-utility model

The economic evaluation was carried out using a theoretical cost-utility analysis framework, using a Markov model structure (cohort health state transition model) [27]. Patients entered the model at commencement of therapy with certolizumab pegol or a comparator. Two certolizumab pegol regimes were analysed: certolizumab pegol (400 mg administered on weeks 0, 2 and 4, then 200 mg every 2 weeks) in combination with methotrexate or as monotherapy. Comparators considered in the analysis were TNFα inhibitors licensed and recommended in Spain in 2009. These included etanercept (25 mg twice weekly), adalimumab (40 mg every 2 weeks or 40 mg weekly), infliximab (3 or 5 mg/kg at week 0, 2, 6 and every 8 weeks thereafter), and etanercept or adalimumab monotherapies.

The population entering the model consisted of patients that had active RA (defined as a disease activity score [DAS28] >5.1, confirmed on at least two occasions a month apart) and had failed to respond adequately to methotrexate. Baseline characteristics were reflective of those patients in clinical practice who are eligible for treatment with certolizumab pegol.

**Fig. 1:**
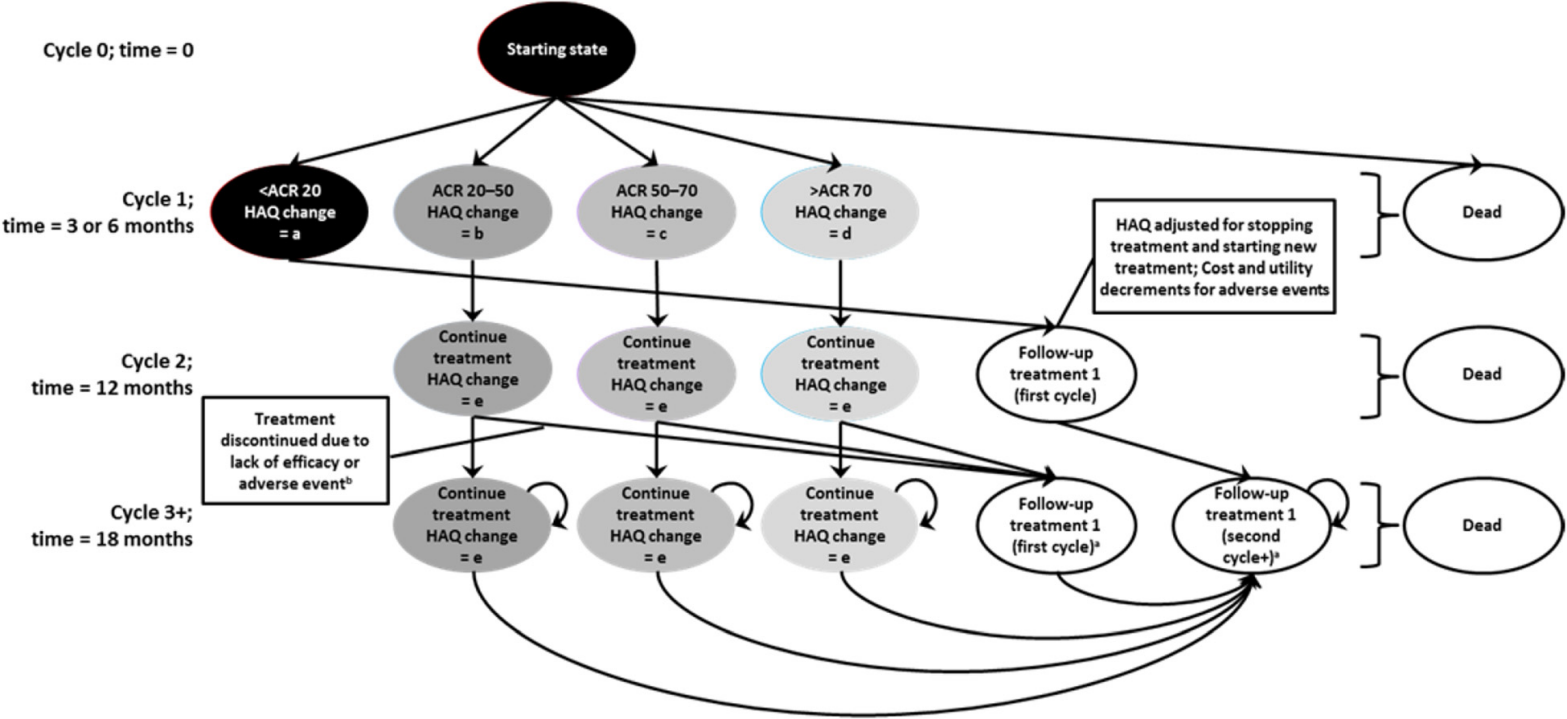
Structure of the Markov model. ^a^ Follow-up treatment states: duplicated for each follow-up treatment. Patients not responding in the first 6 months of follow-up treatment move to the next treatment in the sequence. ^b^ Reason for discontinuation (lack of efficacy or adverse events) determined by the probabilities after leaving treatment health state

The model was developed with a 6-months or a 3-months cycle, depending on when the clinical response is assessed (Fig. 1). At the end of the first cycle, patients were assigned to 1 of 4 response groups, defined according to American College of Rheumatology (ACR) criteria: no response, ACR20, ACR50 or ACR70 response. In patients with an inadequate response (no ACR20 response), treatment was discontinued; only patients who obtained an adequate response in the first time step continued on to the modelled initial therapy. Mortality rates are also assumed during the first cycle of the model [28]. At the end of the next and following cycles, patients may have remained in the same Markov treatment health state; discontinued treatment due to lack of efficacy or due to an adverse event; or died. Patients who discontinued treatment were assumed to have moved on to alternative therapies. On discontinuation of certolizumab pegol or the comparator treatment (adalimumab, etanercept or infliximab), patients may have received the following sequence of conventional DMARD as follow-up therapy: sulfasalazine, leflunomide, gold sodium thiomalate, hydroxychloroquine, azathioprine, cyclosporine, and penicillamine. Upon discontinuation of the last treatment in the sequence, patients received palliative therapy.

After the first 12 months, cycle duration was six months to reflect monitoring frequency recommendations by the Spanish Rheumatology Society [7], the National Institute for Health and Clinical Excellence (NICE) and the British Society of Rheumatology (BSR) [29–31].

Incremental cost-effectiveness ratios (ICERs) are presented, representing the incremental costs necessary to achieve an additional QALY with certolizumab pegol versus the comparator selected.

### Clinical effectiveness estimates and model assumptions

The natural history of the disease and the data on use of resources were derived from various sources, including previous economic evaluation [32]. Treatment duration was obtained from a study with over 2300 patients treated with a TNFα inhibitor over 9 years, which showed that the median treatment duration for TNFα inhibitors was 37 months (3.08 years) [33]. The duration of treatment with small-molecule DMARDs was taken from Chen et al. [32], and all-cause mortality rates for the general population were obtained from published age- and gender-specific mortality rates in Spain [34], adjusted according to Health Assessment Questionnaire-Disability Index (HAQ-DI). The starting mortality rate in cycle 1 is adjusted to the age and gender distribution of the model population and adjustment is made in each model cycle to represent the increased risk of death as patients become older. The base case estimate of relative risk of death of 1.330 per HAQ unit (95 % CI 1.099 to 1.610) is taken from a 35 year cohort study of 3501 RA patients in Canada [35].

Since there were no head-to-head studies directly comparing certolizumab pegol efficacy to that of the other anti-TNF agents, the relative efficacy of the comparators was estimated via an indirect analysis of data from certolizumab pegol studies (RAPID 1 [16, 17], RAPID 2 [36] and FAST4WARD [14]) and from published literature identified through a systematic review [32] included studies of all biological DMARDs published up to April 2009. Medline, Embase and the Cochrane Library (NHSEED) were searched to identify studies of certolizumab pegol, adalimumab, etanercept and infliximab in patients with rheumatoid arthritis. Other forms of arthritis were excluded as were non-English language studies. Studies of the following design were included: economic evaluations piggy-backed on to a clinical trial; cost-consequence, cost-benefit, cost-effectiveness, cost-utility and cost-minimisation analyses; studies in which the comparator was a biological DMARD or a conventional DMARD; and studies that reported quality of life and cost estimates, cost estimates or cost-effectiveness.

Abstracts were first screened by two independent reviewers and any disagreements resolved by a third reviewer. Duplicate citations and those which did not match the eligibility criteria were excluded. Full-text copies of all included and ambiguous studies were obtained. Data from all included studies were extracted independently by two reviewers, and any disagreements when extracted data were compared resolved by a third reviewer. Most data came from trials of biological DMARDs versus methotrexate or placebo. Two direct trials comparing etanercept with infliximab as combination therapies with methotrexate were excluded from the indirect analysis due to their small patient numbers.

RAPID 1 (52-weeks) and RAPID 2 (24-weeks) were both phase III, multicentre, double-blind, randomised placebo-controlled trials evaluating certolizumab pegol 400 mg at weeks 0, 2 and 4 followed by 200 mg or 400 mg plus methotrexate every 2 weeks, or placebo plus methotrexate, in patients with active RA (*n* = 982 and 619, respectively) [16, 17, 36]. Similarly, FAST4WARD was a 24-week, multicentre, double-blind, placebo-controlled phase III study, evaluating certolizumab pegol 400 mg monotherapy every 4 weeks or placebo (*n* = 220) [14]. ACR20 response rate at week 24 was the primary endpoint in all three studies (and co-primary in RAPID 1 study).

In addition to the RAPID 1 and RAPID 2 trials, data for the analysis of combination therapies, with respect to ACR responses at 3 months, were provided by 4 studies. These included one study of adalimumab [37], two of etanercept [38, 39] and one of infliximab [40]. Data on ACR response rates at 6 months were derived from 6 studies, including three studies of adalimumab [9, 37, 41], one of etanercept [39] and two of infliximab [42, 43]. All studies were randomised, double-blind, placebo-controlled studies, except for the study by Westhovens et al. [43], in which patients in the placebo group crossed over to receive active treatment between weeks 22–46.

Data for the analysis of monotherapies were provided by the FAST4WARD study and 3 additional studies each for ACR response rates at 3 and 6 months. The studies from which 3-month ACR response rates were derived included two studies of adalimumab [44, 45] and one study of etanercept [46]; and 6-month ACR response rates were derived from two studies of adalimumab [44, 47] and one of etanercept [46]. All studies were randomised, double-blind, placebo-controlled studies. Estimated ACR response rates, i.e. probabilities of transition of the model, for all agents following the indirect comparison are shown in Table 1.

**Table 1:** Probabilities of transition of the model. Absolute effectiveness: American College of Rheumatology (ACR) response rates (%) at 3 and 6 months estimated from published clinical studies (references cited are the clinical studies upon which the estimates were based)

	3 months			6 months		
	ACR20	ACR50	ACR70	ACR20	ACR50	ACR70
Combination therapies						
MTX [9, 17, 36–43, 61–63]						
	21.5	7.2	2.0	24.2	9.7	3.7
CZP + MTX [16, 17, 36]						
	71.1	35.9	21.6	77.2	49.2	28.2
ADA + MTX [9, 37, 41]						
	70.8	na	na	61.0	41.8	19.7
ETA + MTX [38, 39]						
	66.4	61.1	23.7	68.5	66.4	30.7
IFX + MTX [40, 42, 43]						
	58.6	27.0	19.6	48.2	26.1	11.3
Monotherapies						
Placebo [14, 44–47]						
	14.0	3.2	1.1	13.1	5.7	1.0
CZP [14]						
	53.3	45.1	11.6	55.9	31.4	12.3
ADA [44, 45, 47]						
	55.3	25.7	16.4	39.0	18.8	8.5
ETA [46]						
	46.2	21.4	5.1	62.0	42.3	12.9

*ADA* adalimumab, *CZP* certolizumab pegol, *ETA* etanercept, *IFX* infliximab, *MTX* methotrexate, *na* not available

Data regarding the safety of biological DMARDs varied in the published literature, in terms of the way it is reported and analysed, making it difficult to make indirect comparison between different treatments. However, since safety profiles for all TNF inhibitors, including certolizumab pegol, appear to be similar, costs and results associated with adverse events were not explicitly included in the model.

Resource use data were taken from published literature and unit costs (drug acquisition and administration, monitoring and resources) were taken from official Spanish sources or published references [48, 49]. Treatment unit costs are shown in Table 2 and resource unit costs are shown in Table 3. The cost of methotrexate was assumed to be zero and indirect costs were not included in the model. Costs per HAQ category were taken from a cohort study in Sweden and the United Kingdom [50].

**Table 2:** Treatment unit costs in 2009 [48, 49]

Intervention	Route of administration	Cost (€)	Presentation	Strength (mg)
TNF inhibitor				
CZP	SC inj	474	Pre-filled syringe	200
IFX	IV inf	536.28	Vial	100
ADA	SC inj	514.15	Pre-filled syringe	40
ETA	SC inj	118.40	Pre-filled syringe	25
Conventional DMARDs				
MTX	Oral	2.11	50 tablets	3
Azathioprine	Oral	5.78	50 tablets	50
Cyclosporine	Oral	65.90	30 tablets	100
Auranofin/gold sodium thiomalate	IM inj	6.73	1 vial	50
Hydroxychloroquine	Oral	7.33	30 tablets	200
Leflunomide	Oral	57.59	30 tablets	20
Penicillamine	Oral	6.75	30 tablets	250
Sulfasalazine	Oral	2.38	50 tablets	500
Palliative care	–	0	–	–
Methylprednisolone	IV inf	1.59	1 vial	40

*ADA* adalimumab, *CZP* certolizumab pegol, *ETA* etanercept, *IFX* infliximab, *IM* intramuscular, *INF* infusion, *INJ* injection, *IV* intravenous, *MTX* methotrexate, *SC* subcutaneous

**Table 3:** Resource unit costs in 2009 [64]

Resource	Cost (€)	Description
Appointments with healthcare personnel and personnel time		
Primary care physician	26.78	General medical appointment
Nurse (outpatient clinic)	23.77	Nursing appointment in general medicine (planned appointment)
Hospital nurse	13.19	Cost per hour nursing auxiliary, XHUP (total)
Rheumatologist	99.94	Rheumatology
Hospital pharmacist	105.66	Treatment rheumatology day hospital (drug and material cost will be added)
Administration of IV medication in day hospital	232.80	Day hospital: nursing (drug infusion lasting over 2 h)
Analysis		
Complete blood count	6.37	Complete blood count
Sedimentation rate	3.56	Sedimentation rate
Clinical chemistry profile	6.31	Blood clinical chemistry
Urinalysis	4.00	Urinalysis
Chest X-ray	11.61	Chest X-ray

*IV* intravenous, *XHUP* Xarxa d’Hospitals d’Utilització Pública [Catalan Public Hospitals Network]

**Fig. 2:**
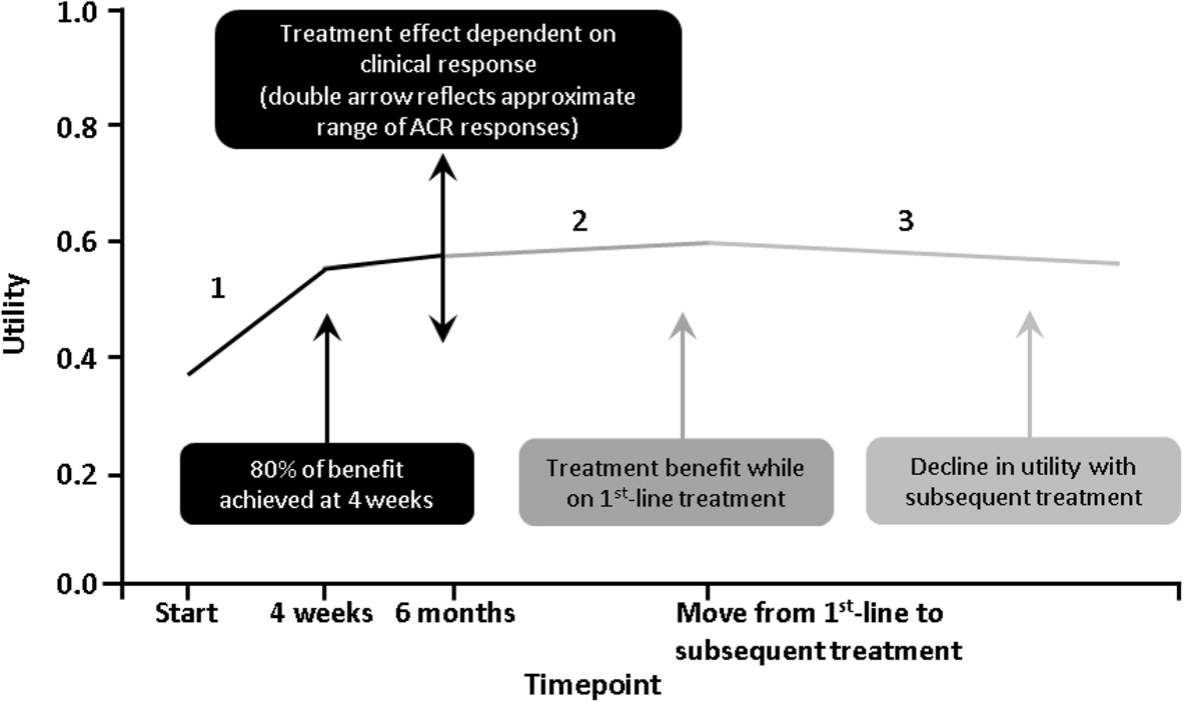
Utilities modelling

Health effects were measured using the EuroQol Group 5 Dimension self-report questionnaire (EQ-5D). Upon entry into the model, the patient population was assigned a mean pre-treatment utility score of 0.38, derived from the EQ-5D data collected in the certolizumab pegol RAPID 1 and 2 trials [17, 36]. Over the first 6 months of initial treatment, patients were assigned an average change in the derived EQ-5D utilities which was dependent on their response category (Fig. 2). The magnitude of the change in EQ-5D utilities was estimated from the patient-level data of the certolizumab pegol trials by ANACOVA regression analysis and the effect is assumed to be the same for all comparators. The regression models were fitted with age, gender, baseline EQ-5D utilities, disease duration, number of previous conventional DMARDs and anti-CCP antibody status as covariates. Default estimates are adjusted for the selected analysis population and are varied in probabilistic sensitivity analyses. Regression coefficients were then used to calculate the change in utility. The base-case analysis also assumes that 80 % of the change over the first 6 months is achieved by week 4. Patients continuing treatment continue to improve over the year following the initial 6 months of treatment, but at a much smaller rate, as the majority of health benefit has been gained by this point (Fig. 2). This assumption was validated by expert clinicians who agreed that it was likely that patients would continue to improve beyond the first round of direct clinical improvements as quality of life impact then becomes more important. Again, it is assumed that certolizumab pegol and all comparators would perform similarly. Changes in HAQ-DI scores with time on treatment are estimated directly from the certolizumab pegol trials using repeated measures analyses and then mapped to EQ-5D utility benefit in the model through the Bansback conversion factor (ΔEQ-5D utility = −0.2102 ΔHAQ) [51]. Patients discontinuing treatment were assigned a decrease in utility equal to that applied for the initial response to treatment and an increase in utility as for the initial response to the first-line treatment, to account for the benefit of the treatment to which the patient moves. Thus the model does not favour interventions from which there is low discontinuation, since the benefit of initial treatment is replaced by a benefit of follow-up treatments. For the follow-on alternative treatments (conventional DMARDs or palliation) the model assumes a decline in the health state over time (Fig. 2).

### Base-case analysis

The base-case analysis was conducted from the perspective of the Spanish National Health System (Sistema Nacional de Salud) and included the following direct healthcare costs: treatment (unit cost, administration, monitoring); hospital costs (outpatient and inpatient); costs of primary-health and specialist medical appointments. The base case analysis assumed a clinical response at 6 months and was conducted over a lifetime horizon (set at 45 years). In this model, for drugs for which the dose is adjusted for patient weight (abatacept, infliximab, azathioprine and cyclosporine), the weight distribution of the RA population in certolizumab pegol trials was used to calculate the percentages of patients receiving a specified number of vials; a patient-fixed average weight of 81.4 kg was assumed. An annual discount rate of 3.5 % was applied for costs and outcomes [52–54]. The analysis assumed a cost per unit approach (i.e. assumes unused drug was wasted). The cost year was 2009, with an inflation rate of 3 % for 2009 onwards. Annual inflation rates for the period 1997 to 2009 were taken from the official statistics published by the Instituto Nacional de Estadística (the Spanish National Statistics Institute) [55].

ICERs were evaluated against a €30,000/QALY willingness-to-pay (WTP) threshold [56–58].

### Sensitivity analyses

Univariate sensitivity analyses were conducted by varying different model parameters including ACR response time of 3 months, costing method (per mg), HAQ-DI measurements for improvements in quality of life instead of EQ-5D, time horizons, discount rate for health costs and outcomes, an assumed association of zero between mortality and HAQ-DI, variation (between 0 and 100 %) in the patient's rate of deterioration on the HAQ-DI scale after discontinuing treatment, baseline HAQ scale score, the annual progression on the HAQ scale for the first-line treatment or continuation treatment.

Probabilistic sensitivity analyses were conducted for each probabilistic sensitivity analysis, 1000 simulations were generated using base case assumptions and parameter variability summarised in Table 4. The variables altered were: clinical effectiveness, mean age, baseline mean HAQ-DI score, number of previous DMARDs, disease duration and antibody status (modelled using a normal distribution), gender (using a beta distribution) and patient weight (using a certolizumab pegol-related cumulative distribution function). All other parameters were held constant. From the results of these simulations, cost-effectiveness planes and cost-effectiveness acceptability curves were drawn.

**Table 4:** Parameters varied through probabilistic sensitivity analysis

Parameter	Simulation	Source
Clinical effectiveness	The log odds of response were simulated from a Normal distribution with mean and standard deviations derived from the CIs of the network meta-analysis (transformed to a log odds scale).	Indirect analysis results.
Association between mortality and HAQ-DI score	The relative risk was simulated from a Lognormal distribution with parameters implied by the point estimate (1.330) and its confidence interval (1.099 to 1.610)	Wolfe et al. [35]
Age (years)	Normal distribution defined by the mean (52.165) and the standard error (51.893 to 52.4378)	RAPID 1, RAPID 2 and FAST4WARD
Gender	Beta distribution defined by N (1821) and n(1506)	RAPID 1, RAPID 2 and FAST4WARD
Weight	A cumulative distribution function derived from CZP-related data.	RAPID 1, RAPID 2 and FAST4WARD
Baseline HAQ-DI score	Normal distribution defined by the mean (1.624) and its confidence interval (1.610 to 1.638)	RAPID 1, RAPID 2 and FAST4WARD
Number of previous DMARD	Normal distribution defined by the mean (2.258) and its confidence interval (2.207 and 2.308)	RAPID 1, RAPID 2 and FAST4WARD
Disease duration	Normal distribution defined by the mean (6.557) and its confidence interval (6.351 and 6.763)	RAPID 1, RAPID 2 and FAST4WARD
Anti-CCP antibody positive	Normal distribution defined by the mean (1.676) and its confidence interval (1.611 and 1.741)	RAPID 1, RAPID 2 and FAST4WARD
Anti-CCP antibody negative	Normal distribution defined by the mean (1.621) and its confidence interval (1.606 and 1.635)	RAPID 1, RAPID 2 and FAST4WARD
Utility weight	Sampled from a randomized percentage of population (mean 0.380 and confidence interval 0.372 and 0.388)	RAPID 1 and RAPID 2

## Results

### Base-case analysis

#### Combination therapies

Certolizumab pegol plus methotrexate was the most cost-effective therapy when compared with other biologic TNF inhibitors plus methotrexate, at €30,000/QALY willingness-to-pay (WTP) threshold [56–58]. Certolizumab pegol + methotrexate dominated (most efficient as assessed through QALYs and less expensive) adalimumab (weekly), etanercept, infliximab 5 mg/kg, combination therapies. Combination certolizumab pegol plus methotrexate was cost-effective versus adalimumab (every 2 weeks) and infliximab 3 mg/kg in combination with methotrexate, with estimated ICERs of €12,346/QALY and €15,414/QALY, respectively (see Table 5).

**Table 5:** Base case results for the treatment of rheumatoid arthritis in Spain over a lifetime horizon (45 years)

	Mean costs (€)	Difference in costs vs CZP (€)	Mean QALY	Difference in QALYs vs CZP	ICER vs CZP
Combination therapies					
CZP + MTX	140,971	0	6.578	0	–
ADA + MTX (every 2 weeks)	139,148	1823	6.430	0.148	€12,346
ADA + MTX (weekly)	164,741	−23,770	6.430	0.148	CZP dominant
ETA + MTX	141,197	−226	6.462	0.116	CZP dominant
IFX (3 mg/kg) + MTX	136,961	4010	6.318	0.260	€15,414
IFX (5 mg/kg) + MTX	152,561	−11,590	6.318	0.260	CZP dominant
Monotherapies					
CZP	134,792	0	6.416	0	–
ADA (every 2 weeks)	136,745	−1953	6.216	0.200	CZP dominant
ADA (weekly)	156,223	−21,431	6.216	0.200	CZP dominant
ETA^a^	135,459	667	6.492	0.076	€8,778^a^

*ADA* adalimumab, *CZP* certolizumab pegol, *ETA* etanercept, *ICER* incremental cost-effectiveness ratio, *IFX* infliximab, *MTX* methotrexate, *QALY* quality-adjusted life years

^a^Incremental Analysis is for ETA versus CZP and not the other way round

#### Monotherapies

The analysis indicated that certolizumab pegol monotherapy was the most effective (as measured through QALYs) and less expensive compared with adalimumab (weekly or every 2 weeks). Certolizumab pegol monotherapy was also less expensive, but had comparable health gains when compared with etanercept monotherapy (6.416 QALYs vs 6.492), that lead to an ICER of €8788/QALY (etanercept vs certolizumab pegol). See Table 5.

### Sensitivity analyses

#### Univariate sensitivity analysis

The results of the analysis of sensibility are show in the Tables 6 and 7. The sensitivity analyses indicated that the base case results were robust, with certolizumab pegol remaining the cost-effective treatment at the €30.000/QALY WTP threshold, when changes were applied to the discount rate, economic perspective of the analysis, the drug costing method, the choice of quality of life instrument and the association between HAQ score and mortality. The ICERs were sensitive to changes in the time horizon, timepoint of ACR response, baseline HAQ score, and the rate of deterioration in HAQ-DI scale after discontinuing the treatment.

**Table 6:** One way sensitivity analysis for the ICER of certolizumab pegol vs. monotherapies

Parameter	Base case estimate	Sensitivity estimate	Comparator to certolizumab pegol (incremental cost per QALY gained)		
			Adalimumab (every 2 weeks)	Adalimumab (weekly)	Etanercept
Base case results			CZP dominates	CZP dominates	8,778^a^
Time horizon	Lifetime	5 years	29,944	CZP dominates	5,537^a^
		10 years	11,327	CZP dominates	8,033^a^
Discount rate	Costs and QALYs 3.5 %	Costs 1.5 % and QALYs 1.5 %	CZP dominates	CZP dominates	11,131^a^
		Costs 1.5 % and QALYs 6 %	CZP dominates	CZP dominates	13,572^a^
		Costs 6 % and QALYs 1.5 %	CZP dominates	CZP dominates	5,481^a^
		Costs 6 % and QALYs 6 %	CZP dominates	CZP dominates	6,683^a^
Inflation	3.0 %	0 %	CZP dominates	CZP dominates	11,017^a^
ACR response	6 months	3 months	17,919^a^	593.646^a^	ETA dominates
Baseline HAQ score	1.6	1	206,601^a^	1.735.397^a^	88,132
		2.5	16,918	CZP dominates	11,394^a^
Rebound assumption, back to baseline	100 %	50 %	21,616	CZP dominates	61,234^a^
Perspective	SNS	Societal	CZP dominates	CZP dominates	ETA dominates
Drug costing	Per mg	Per unit	CZP dominates	CZP dominates	7,928^a^
Principle QoL instrument	EQ-5D	HAQ DI	2457	CZP domina	380^a^
Association between HAQ DI and mortality	RR of 1.33 per HAQ DI increment	No association (RR of 1)	CZP domina	CZP domina	11,418^a^
Administration Cost of IV medication in day hospital	214,54 €	+20 % (257,44 €)	subcutaneous injections	subcutaneous injections	subcutaneous injections
		−20 % (171,63 €)	subcutaneous injections	subcutaneous injections	subcutaneous injections

^a^ICER is of adalimumab or etanercept against CZP, rather than vice versa, as elsewhere

**Table 7:** One way sensitivity analysis for the ICER of certolizumab pegol + MTX vs. combination therapies

Parameter	Base case estimate	Sensitivity estimate	Comparator to certolizumab pegol (incremental cost per QALY gained)				
			Adalimumab (every 2 weeks) + MTX	Adalimumab (weekly) + MTX	Etanercept + MTX	Infliximab (3 mg/kg) + MTX	Infliximab (5 mg/kg) + MTX
Base case results			12,346	CZP dominates	CZP dominates	15,414	CZP dominates
Time horizon	Lifetime	5 years	36,676	CZP dominates	406,743	27,472	CZP dominates
		10 years	29,024	CZP dominates	87,096	24,861	CZP dominates
Discount rate	Costs and QALYs 3.5 %	Costs 1.5 % and QALYs 1.5 %	7736	CZP dominates	CZP dominates	12,924	CZP dominates
		Costs 1.5 % and QALYs 6 %	9815	CZP dominates	CZP dominates	15,596	CZP dominates
		Costs 6 % and QALYs 1.5 %	13,621	CZP dominates	6372	14,747	CZP dominates
		Costs 6 % and QALYs 6 %	17,281	CZP dominates	11,624	17,796	CZP dominates
Inflation	3.0 %	0 %	14,385	CZP dominates	CZP dominates	CZP dominates	CZP dominates
ACR response	6 months	3 months	996^a^	78.117^a^	CZP dominates	5461	CZP dominates
Baseline HAQ score	1.6	1	32,708^a^	565.932^a^	8459	50,134	123,991^a^
		2.5	13,742	CZP dominates	46,630	14,677	CZP dominates
Administration Cost of IV medication in day hospital	214,54 €	+20 % (257,44 €)	subcutaneous injections	subcutaneous injections	subcutaneous injections	13,309	CZP dominates
		−20 % (171,63 €)	subcutaneous injections	subcutaneous injections	subcutaneous injections	17,519	CZP dominates
Rebound assumption, back to baseline	100 %	50 %	58,330	CZP dominates	106,252	46,733	25,385
Perspective	SNS	Societal	CZP dominates	CZP dominates	CZP dominates	CZP dominates	CZP dominates
Drug costing	Per mg	Per unit	12,821	CZP dominates	CZP dominates	29,538	CZP dominates
Principle QoL instrument	EQ-5D	HAQ DI	9791	CZP dominates	28,013	8709	CZP dominates
Association between HAQ DI and mortality	RR of 1.33 per HAQ DI increment	No association (RR of 1)	7766	CZP dominates	CZP dominates	12,608	CZP dominates

^a^ICER is of etanercept + MTX, infliximab + MTX or rituximab + MTX versus CZP + MTX, rather than vice versa, as elsewhere

#### Probabilistic sensitivity analyses

**Fig. 3:**
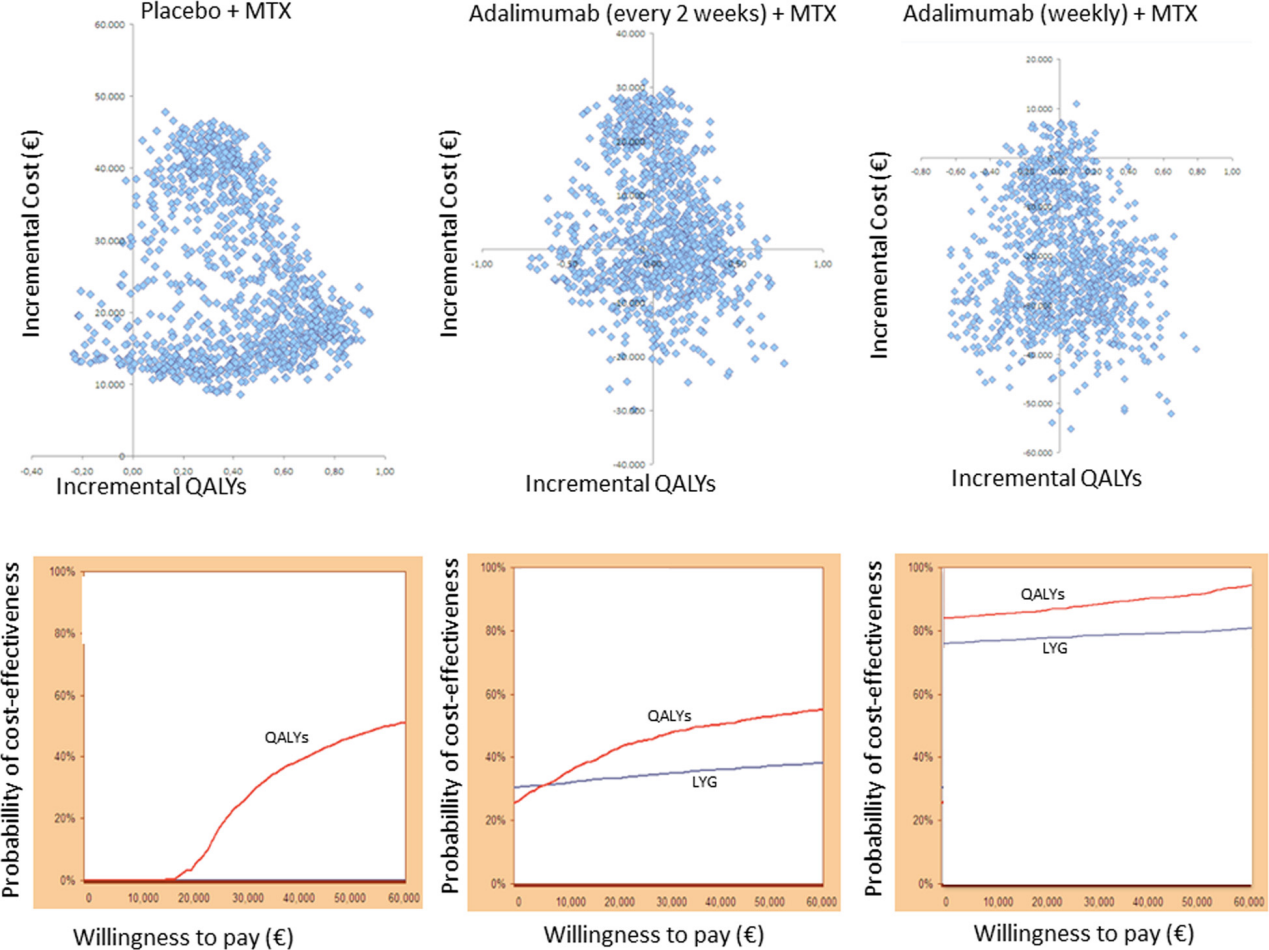
Plane of incremental costs (£) vs. incremental QALYs and Cost-effectiveness acceptability curve: Certolizumab pegol + MTX vs MTX; Certolizumab pegol + MTX vs Adalimumab (every 2 weeks) + MTX and Certolizumab pegol + MTX vs Adalimumab (weekly) + MTX

**Fig. 4:**
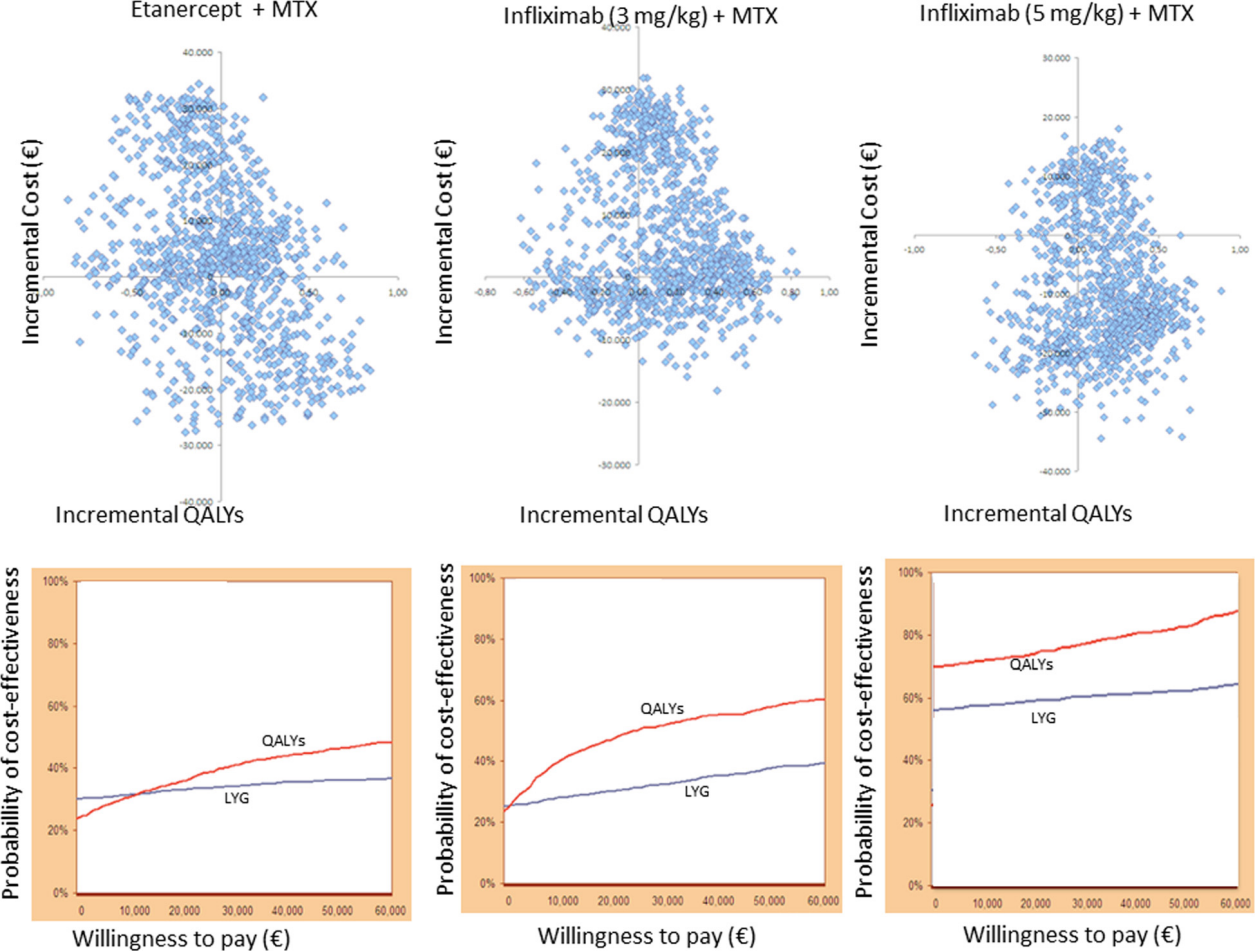
Plane of incremental costs (£) vs. incremental QALYs and Cost-effectiveness acceptability curve: Certolizumab pegol + MTX vs Etanercept + MTX; Certolizumab pegol + MTX vs Infliximab (3 mg/kg) + MTX and Certolizumab pegol + MTX vs Infliximab (5 mg/kg) + MTX

Cost-effectiveness planes for the PSA of certolizumab pegol vs. other therapies with the ACR six month definition of response are shown in Figs. 3 and 4. The probabilistic sensitivity analysis indicated that at €30.000/QALY WTP, certolizumab pegol plus methotrexate has the highest probability of being cost-effective against adalimumab (weekly) and infliximab (5 mg/kg), in 91 % and 78 % of the cases, respectively. When compared with adalimumab every 2 weeks, etanercept and infliximab (3 mg/kg), and certolizumab pegol had almost equal probability of being cost-effective (42 to 48 % at a €30.000/QALY WTP threshold).

## Discussion

The cost utility of certolizumab pegol compared with other TNF inhibitors available in Spain in 2009 and administered alone or in combination with methotrexate in patients with moderate-to-severe RA who have had an inadequate response to methotrexate alone has been estimated. Evaluated against a €30,000/QALY WTP threshold and over a lifetime horizon, certolizumab pegol administered in combination with methotrexate was a dominant therapy compared with other TNF inhibitor combination therapies (weekly adalimumab, etanercept, and infliximab 5 mg/kg), and cost-effective versus 2-weekly adalimumab and infliximab (3 mg/kg) combination therapies.

Certolizumab pegol monotherapy was dominant versus weekly or 2-weekly adalimumab monotherapy and was also less expensive, but had comparable health gains when compared with etanercept monotherapy. Probabilistic sensitivity analyses confirmed the cost-effectiveness of certolizumab pegol + methotrexate combination therapy. Univariate sensitivity analyses showed that ICERs for certolizumab pegol administered with or without methotrexate were robust to changes in the majority of variables analysed, and that ICERs were sensitive to changing the time horizon, timepoint of ACR response, baseline HAQ score, and the rate of deterioration in HAQ-DI scale after discontinuing the treatment.

Comparison with other studies is difficult since most of the studies published to date support the cost-effectiveness of TNF inhibitors as a second-line strategy in patients who fail to respond to non-biological DMARDs, but relative evaluation of TNF inhibitors using indirect cost-effectiveness analyses are lacking. Only one study, evaluating the relative cost-effectiveness of the five current FDA-approved TNFα inhibitors in combination with methotrexate for the treatment of patients with moderate-to-severe active RA and with moderate or no response to methotrexate monotherapy from a US health payer perspective, has been recently published [59]. The study used Bayesian methods to determine the relative probabilities of achieving an ACR50 clinical response for each TNF inhibitor and Markov modelling, in which patients who achieved ACR50 criteria continued to receive combination therapy but those who did not switched to tocilizumab. Results of the study showed certolizumab pegol + methotrexate to be the second most cost-effective TNF inhibitor after etanercept + methotrexate, dominating infliximab, adalimumab and golimumab combination therapies at a WTP threshold of US$139,143/QALY.

Whilst estimation of relative effectiveness of the various biological DMARDs using ACR response data from only randomised, double-blind trials with methotrexate and/or placebo as controls helped to ensure the quality of these data, the lack of head-to-head studies, necessitating indirect analysis of ACR response data, is acknowledged as a limitation of our study. Also, although similar incidences of adverse events between the available biological DMARDs may be generally considered to be a reasonable assumption, exclusion of the costs associated with adverse events from the model is another acknowledged limitation of our study. Other limitations include the lack of data sources and cost-utility data (in Spain and worldwide) with which to compare our data, and the limited effectiveness of applying effectiveness information from 6 months, which is >1 treatment cycle.

The implications of our study findings for the public payer in Spain suggest that the Spanish NHS would be adopting the most cost-effective treatment if certolizumab pegol were offered to RA patients in combination with methotrexate instead of alternative TNF inhibitors currently recommended in Spain. Furthermore a recent study, in that equality of effectiveness is supposed, indicated that the addition of certolizumab pegol on the NHS in Spain, would generate large net savings of €10.3 million for the period 2013 to 2017 [60].

## Conclusions

In our study, in terms of QALYs gained, certolizumab pegol was the most effective therapy in combination with methotrexate at the €30,000/QALY willingness-to-pay (WTP) threshold compared with other TNF inhibitors recommended in Spain in 2009 (adalimumab, etanercept and infliximab) for the treatment of patients with active RA who did not respond adequately to DMARDs. In an analysis of monotherapies, certolizumab pegol was more cost-effective versus adalimumab and less expensive with similar health gains versus etanercept (mean QALY 6.416 vs 6.492). Probabilistic sensitivity analyses indicated advantages in efficacy for certolizumab pegol over the other TNF inhibitors. These results indicate that moderate-to-severe RA treatment with certolizumab pegol is an efficient and economically valuable alternative for the Spanish NHS.

## Abbreviations

ACR: American College of Rheumatology
ACR20: American College of Rheumatology 20 % response
ACR50: American College of Rheumatology 50 % response
ACR70: American College of Rheumatology 70 % response
DAS28: Disease Activity Score 28
DMARDs: disease-modifying antirheumatic drugs
EQ-5D: EuroQol Group 5 Dimension
HAQ: Health Assessment Questionnaire
HAQ-DI: Health Assessment Questionnaire-Disability Index
ICERs: incremental cost-effectiveness ratios
LYG: life year gained
QALY: quality-adjusted life year
RA: rheumatoid arthritis
TNF: tumour necrosis factor
WTP: willingness-to-pay
